# Correction: Individualized Homeopathic Treatment and Fluoxetine for Moderate to Severe Depression in Peri- and Postmenopausal Women (HOMDEP-MENOP Study): A Randomized, Double-Dummy, Double-Blind, Placebo-Controlled Trial

**DOI:** 10.1371/journal.pone.0127719

**Published:** 2015-05-04

**Authors:** The PLOS ONE Staff

In the Abstract, the link to the trial registration is incorrect. The correct link is: https://clinicaltrials.gov/ct2/show/NCT01635218. The publisher apologizes for the error.
